# Supplementary material for: Silver Nanoparticles Agglomerate Intracellularly Depending on the Stabilizing Agent: Implications for Nanomedicine Efficacy
Source: Nanomaterials (Basel). 2020 Sep 30;10(10):1953. doi: 10.3390/nano10101953 (PMC7601179; doi:10.3390/nano10101953)
Supplement: Supplementary file 1 [file nanomaterials-10-01953-s001.pdf]

Supplementary Materials

Silver Nanoparticles Agglomerate Intracellularly Depending on the Stabilizing Agent: Implications for Nanomedicine Efficacy

Marina R. Mulenos, Henry Lujan, Lauren R. Pitts and Christie M. Sayes*

[1] Department of Environmental Science, Baylor University, Waco, TX, 76798-7266, United States
Marina_George@baylor.edu (M.R.M.); Henry_Lujan@baylor.edu (H.L.); Lauren_Pitts@baylor.edu (L.R.P.)
* Correspondence: christie_sayes@baylor.edu; Tel: 1-254-710-3469; Fax: 1-254-710-3409

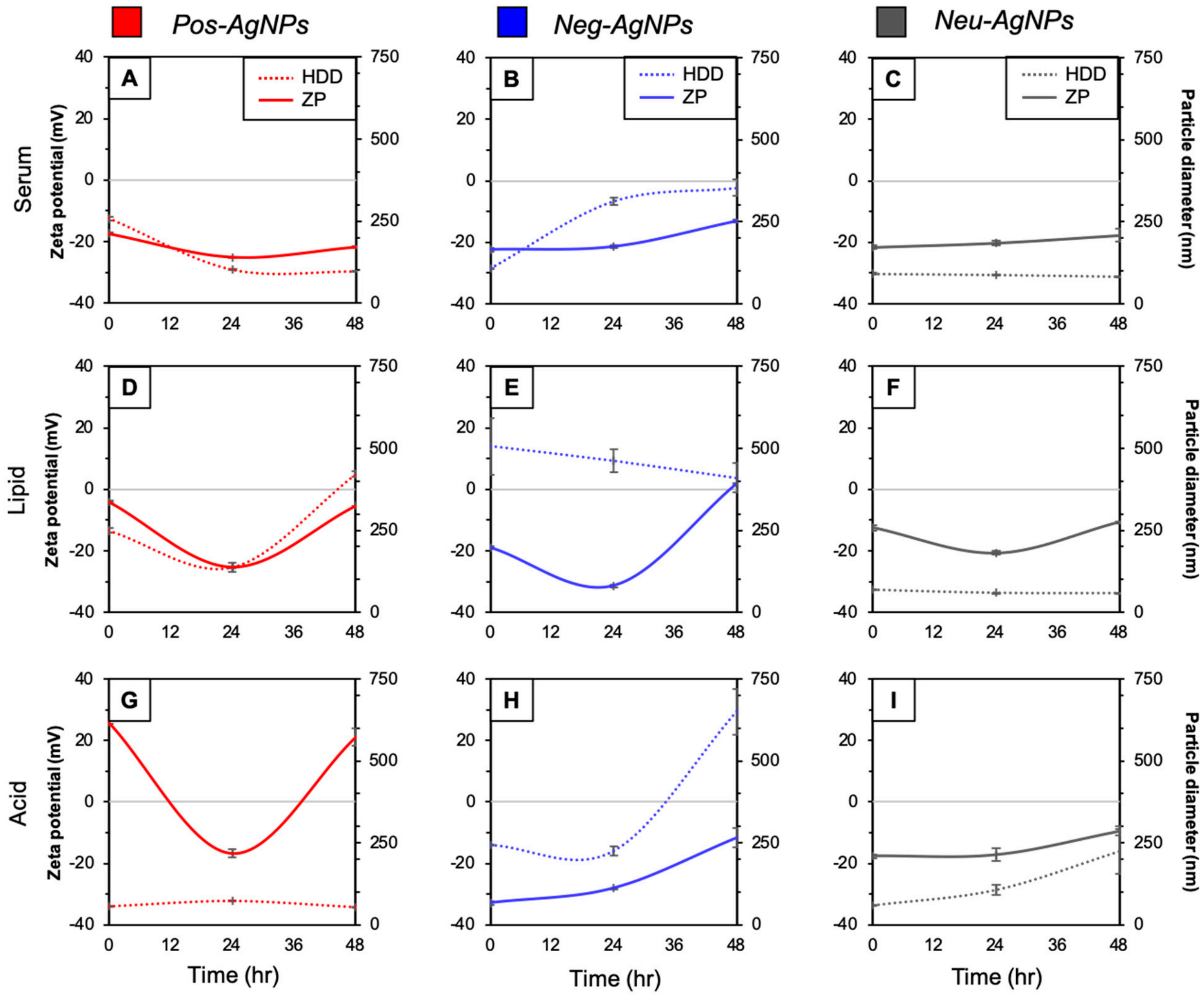

Figure S1. Trends in AgNP HDD and ZP over time showed significant changes among stabilizing agents charge, but not incubation scenario. No trends were found in the Neg-AgNPs (B,E,H) plots.

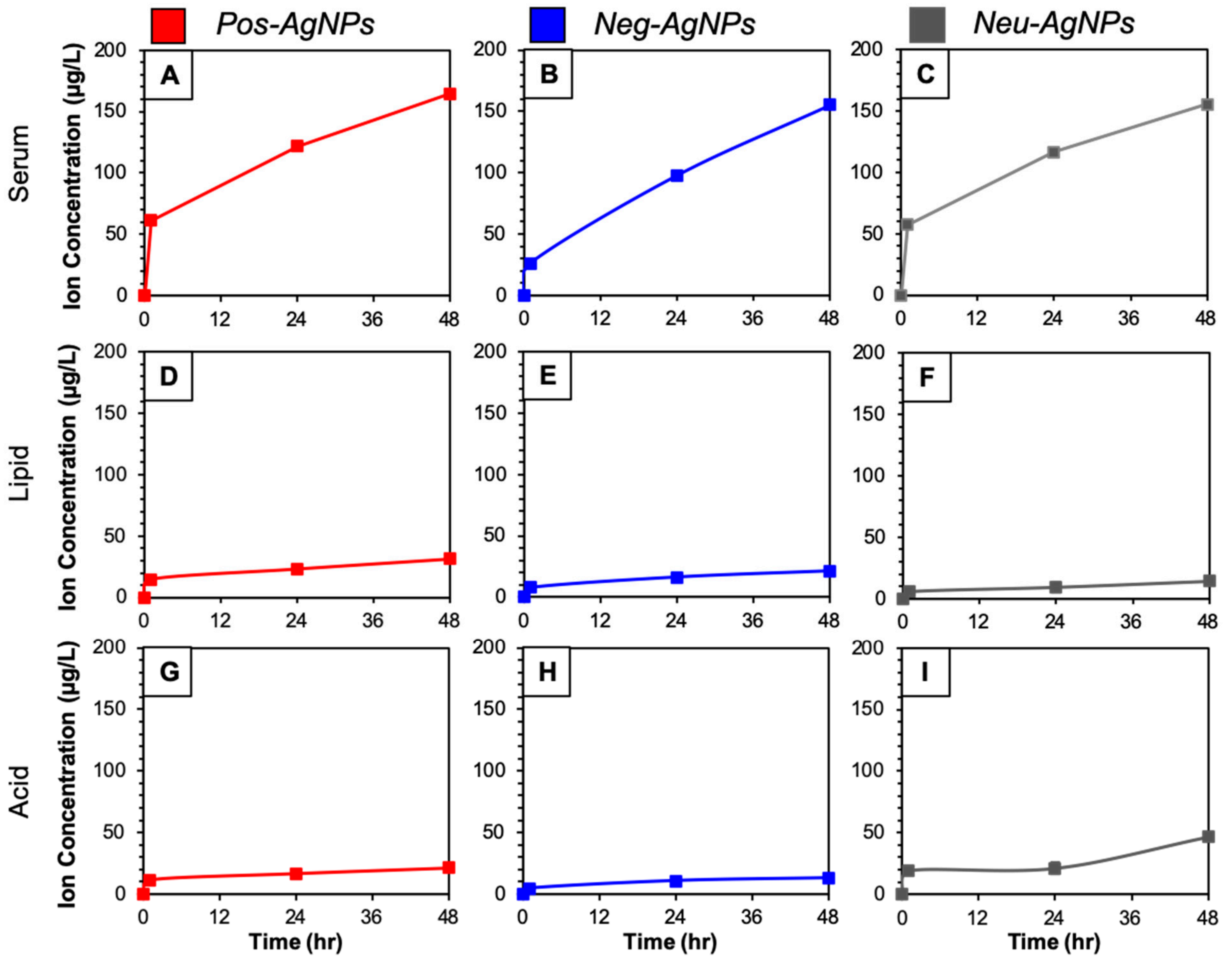

Figure S2. After biotransformation, silver ion dissolution was measured utilizing ICP-MS at 0, 24, and 48 h. Serum protein absorption (A,B&C) produced the highest concentration of dissociated silver for all three time periods tested, which is in contrast to (B) lipid and (C) acid which had negligible dissolution in comparison.

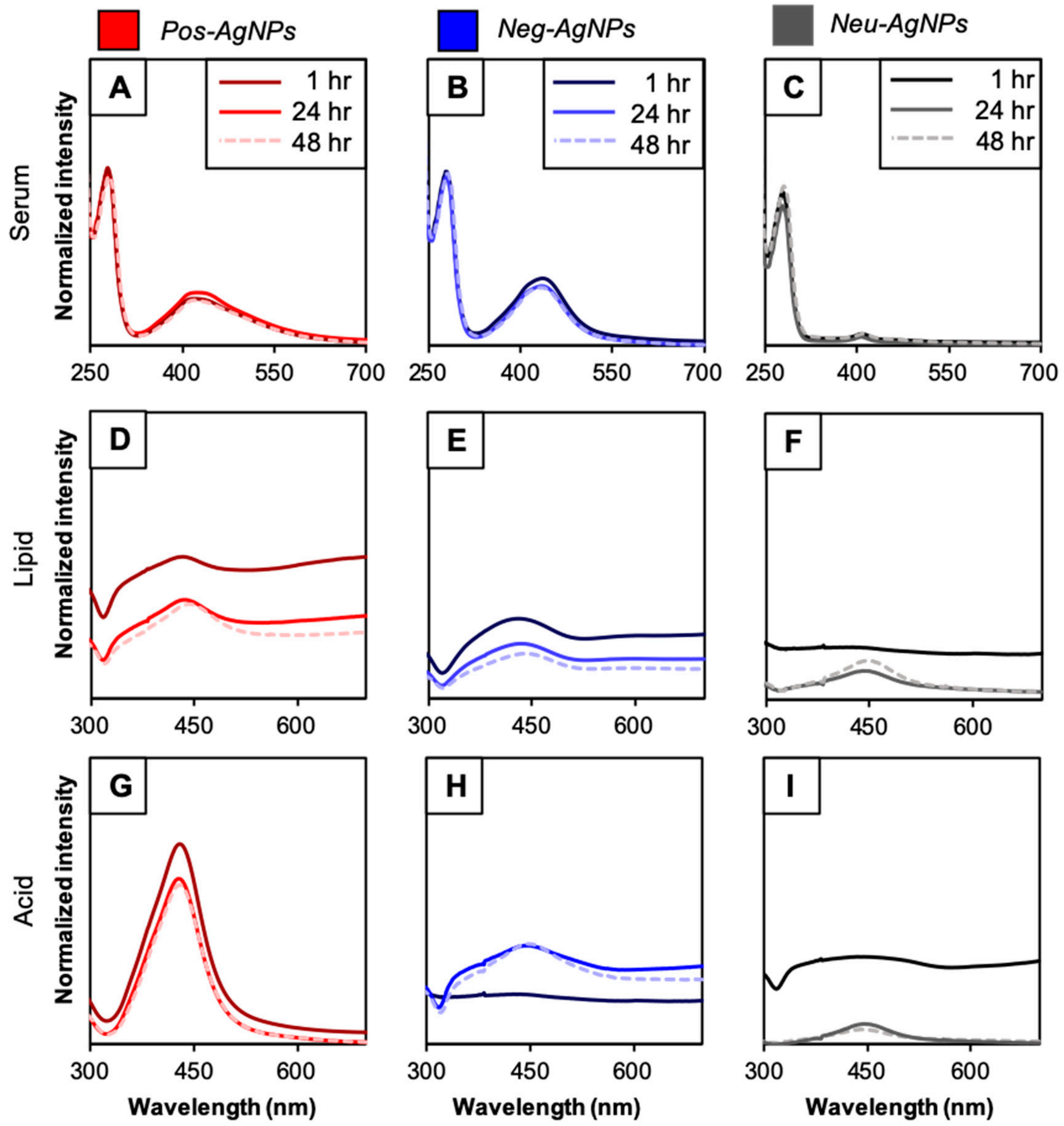

Figure S3. UV-Vis absorbance spectra monitored at 1, 24, and 48 h. timepoints post-incubation. AgNPs change over time after serum incubation indicated by increased absorption compared to the other incubation scenarios (D–I). Neu-AgNPs (F) and digested Neg-AgNPs (H) exhibited a decrease in absorbance. .
